# 

**Published:** 1885-11

**Authors:** 


CONTENTS.

Vol. 32 No. 11

| | PAGE. |
|---|---|
| Bacteria | 1 |
| The Stomach of the Horse | 2 |
| A Wonderful Peruvian Railroad | 3 |
| Pilocarpine for Deafness | 5 |
| Hypodermic Medication | 5 |
| Medicinal Plants in Brazil | 6 |
| The Possible Suspension of Old Age | 6 |
| Remedy for Earache | 9 |
| Erysipelas—Its Rapid Cure | 9 |
| Whooping Cough | 10 |
| Nutritive Value of the Different Parts of the Grain of Wheat | 11 |
| Supposed Dialogue about Temperance | 12 |
| A Saline Intra-venous Injection for the Treatment of Cholera | 13 |
| Copper and Cholera | 14 |
| The Use of Arsenic in Pulmonary Tuberculosis | 15 |
| A Cool Detective | 16 |
| Fashionable Freaks | 17 |
| Natural Death | 17 |
| Educated Girls | 18 |
| One Good Deed | 19 |
| Miscellaneous | 20 |

